# Bibliometric and visual analysis of mesenchymal stem cells in the treatment of osteoporosis based on CiteSpace software

**DOI:** 10.1097/MD.0000000000031859

**Published:** 2022-11-18

**Authors:** Runfang Wang, Yueying Wang, Weiyi Zai, Ning Xu

**Affiliations:** a Medical School of Rehabilitation, Shandong University of Traditional Chinese Medicine, Jinan, China.

**Keywords:** bibliometrics, CiteSpace, mesenchymal stem cells, osteoporosis, visualization analysis

## Abstract

The focus of research in the treatment of osteoporosis (OP) has evolved from promoting bone formation and inhibiting bone resorption to current stem cell therapy. Due to their multipotent differentiation properties, mesenchymal stem cells (MSCs) can repair degenerated bones through transplantation, and have become a new method for the treatment of OP.

**Methods:**

Relevant literatures included in the Web of Science database core collection database from 2012 to 2021 were retrieved. CiteSpace software was used to analyze the cooperative relationship among authors, journals, institutions, and countries, and to analyze the co-citation situation of the literature. And performed co-occurrence analysis, cluster analysis and burst analysis of keywords, draw visual maps and analyzed the results.

**Results:**

A total of 2100 papers were included, and the number of papers published from 2012 to 2021 was on the rise. A total of 484 authors were included, and 176 authors published more than 3 papers. The high-yield authors were mainly represented by YAN JIN and BO GAO. A total of 99 journals were included, and the journal with the most publications was *J BONE MINER RES*. A total of 787 institutions were included, and the institution with the largest number of publications was Shanghai Jiao Tong University. A total of 65 countries were included. The country with the largest number of publications was China, and the United States had the highest centrality. The co-citation analysis of the literature found 2 articles with high citation frequency and high centrality. The main research direction was the mechanism of MSCs in the treatment of osteoporosis. A total of 133 keywords were included, and the hot keywords were osteogenic differentiation, expression, proliferation, bone marrow, etc.

**Conclusions:**

The research hotspots in this field mainly focused on the mechanism of bone regeneration, proliferation and osteogenic differentiation of bone marrow MSCs, and the expression of osteogenic-related genes. The future research trends in this field are predicted to be the mechanism of action of microRNA and long non-coding RNA on MSCs and their relationship with OP, the mechanism of MSCs adipogenic and osteogenic differentiation, and tissue engineering scaffolds applications.

## 1. Introduction

Osteoporosis (OP) is the most common chronic metabolic bone disease characterized by loss of bone strength due to an imbalance between bone resorption and bone formation mechanisms, leading to increased fragility and fractures. The pathophysiological mechanism of this disease includes an insufficient formative response during the remodeling of bone formation, which is an important factor in the pathogenesis of OP.^[1]^ OP can generally be divided into two categories based on the known etiology. One is primary osteoporosis, which usually occurs in elderly and postmenopausal women, and is the most common type of OP. The second is secondary osteoporosis with a clear pathogenic mechanism.^[2]^ The spine, wrist, hip, and humeral head are the main sites of fractures caused by OP, and the negative impact of spine and hip fractures is greater, resulting in back pain, spinal deformity, loss of independence, and even death.^[3]^ At present, in the clinical treatment of OP, drug treatment is more common, mainly including bisphosphonates, estrogen, calcitonin and parathyroid hormone, etc., but these drugs have certain side effects.^[4]^ Therefore, it is very important to explore safer and more effective alternative therapies for the treatment of OP. Mesenchymal stem cells (MSCs) are pluripotent stromal cells that can self-renew and differentiate into mesoderm cells, such as bone, fat, and chondrocytes, and play an important role in the pathogenesis and treatment of OP.^[5]^ The mechanism by which MSCs treat OP is mainly attributed to the paracrine effect. By releasing a large number of functional molecules, MSCs are absorbed by damaged tissues or cells, thereby facilitating angiogenesis, proliferation, inhibiting apoptosis and inflammation, and promoting osteogenesis.^[6]^

CiteSpace software was created by Professor Chen Chaomei of Drexel University in the United States. It is a knowledge management software for bibliometric analysis.^[7]^ By generating a visual knowledge map, CiteSpace explores the research status, research hotspots, research fronts and evolution process of a scientific field, reveals research directions, research stages and frontier characteristics, and finally judges the development trend of this field.^[8]^ So far, CiteSpace software has been used in various fields such as environmental science, education and public health.^[9]^ In this paper, CiteSpace software was used to visually analyze the authors, countries, keywords and other data included in the literature, summarized the research hotspots and development trends in this research field, and provided support and reference for in-depth related research work.

## 2. Materials and methods

### 2.1. Data source and retrieval strategy

#### 2.1.1. Data source.

The Web of Science database core collection (WOSCC).

#### 2.1.2. Search strategy.

The retrieval strategy was “TS = (osteoporosis* OR OP) AND TS = (‘mesenchymal stem cell*’ OR MSC).” The time span was set to 2012 to 2021. The document type was limited to “Article.” The language type was set to “English.” The last search date was 2022-04-20, and a total of 2100 related literatures were obtained.

### 2.2. Methods

The retrieved literature was exported in the format of “full record and cited references” and “plain text,” a total of 2586 articles, named “download_XXX.txt.” After deduplication by Citespace5.8.R3 software, no duplicate literature was found. By manually excluding conference papers, books and other unrelated literature, 2100 articles were finally included. In CiteSpace5.8.R3 software parameter settings, set the time span to 2012-2021, the time division to 1 year, the threshold item to select “Top N” (the value is set to 50), selected “Pathfinder” and “Pruning sliced” networks” as a cut connection method to simplify the network structure and highlight important features. The node type selected authors, institutions, countries, journals, cited documents and keywords for visual analysis, and drew knowledge graphs. The size of each node represents the number of documents. The purple outer circle of a node indicates that the node has high centrality and may be a turning point or key point in the field. The connection between nodes indicates co-occurrence or co-citation relationship, and the thicker the connection is, the closer the connection is.^[10]^ Nodes of centrality are often considered to be turning points or pivot points in a field.^[11]^

## 3. Results

### 3.1. Annual publications

**Figure 1.**
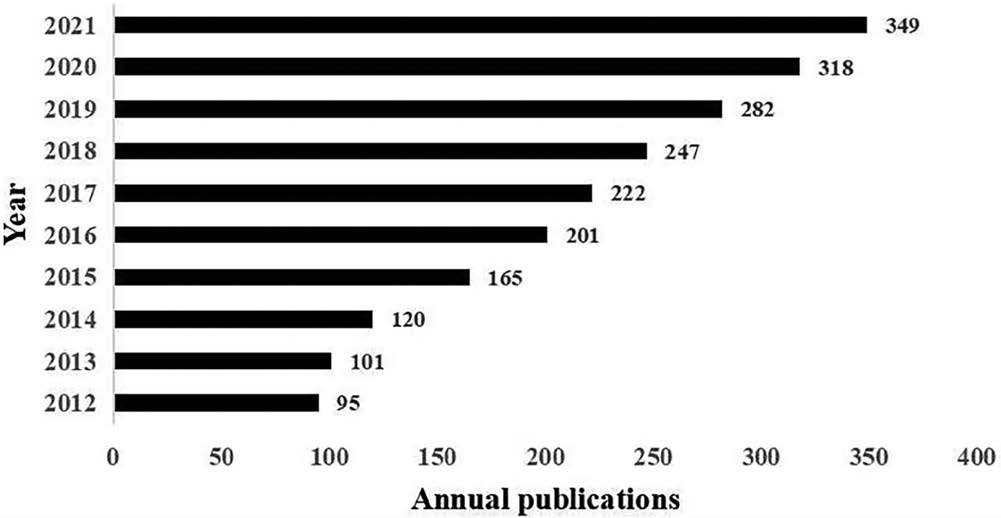
The annual number of publications.

In the end, 2100 papers were included, and a line chart was drawn with the year as the abscissa and the annual number of publications as the ordinate (Fig. 1). As shown in the figure, the number of published papers in this field in general had a steady upward trend from 2012 to 2021. The number of papers published in 2021 is about 3.7 times that of 2012, indicating that research in this field had continued to develop in the past 10 years and had good prospects for development.

### 3.2. Authors

**Figure 2.**
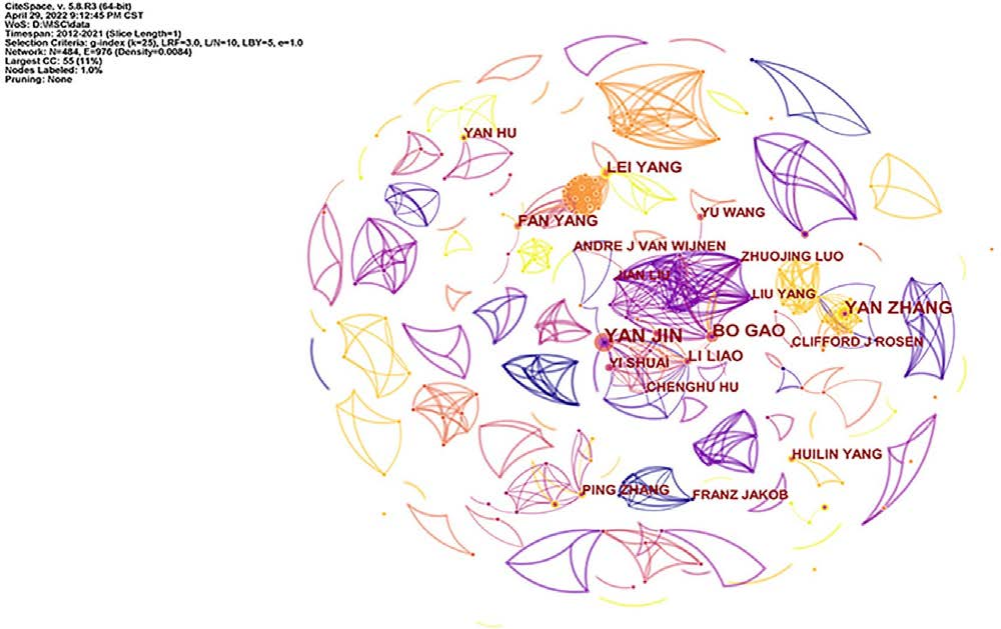
Knowledge map of author collaboration.

By analyzing the cooperation network of authors in this field, a knowledge graph consisting of 484 nodes and 976 connections was obtained, as shown in Figure 2. The authors with the highest number of published papers were YAN JIN (24 papers), YAN ZHANG (19 papers), LEI YANG (16 papers), BO GAO (16 papers) and LI LIAO (14 papers), etc. The author with the highest centrality was BO GAO (0.02) of the Institute of Orthopaedic Surgery, Xijing Hospital, Fourth Military Medical University. Most of the authors with the highest number of publications and centrality were from China, indicating that Chinese scholars’ research in this field has a great influence on the world.

### 3.3. Journals

**Figure 3.**
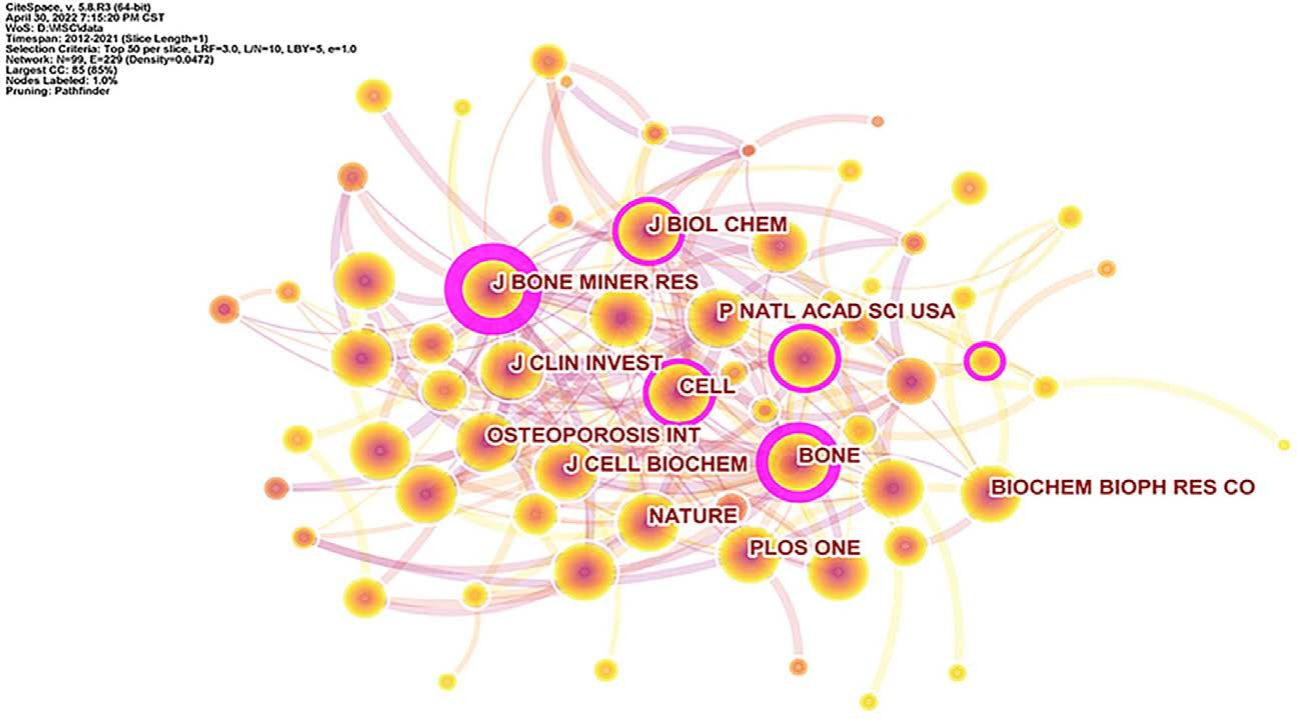
Knowledge map of journal collaboration.

By analyzing the journal cooperation network in this field, a knowledge graph consisting of 99 nodes and 229 connections was obtained, as shown in Figure 3. The top journals in terms of publication volume were J BONE MINER RES (1371 papers), BONE (1316 papers), J BIOL CHEM (988 papers), PLOS ONE (943 papers) and J CELL BIOCHEM (877 papers), etc.; The top journals were J BONE MINER RES (0.48), BONE (0.36), SCI REP-UK (0.16), CELL (0.15) and J BIOL CHEM (0.14), etc.

### 3.4. Institutions

**Figure 4.**
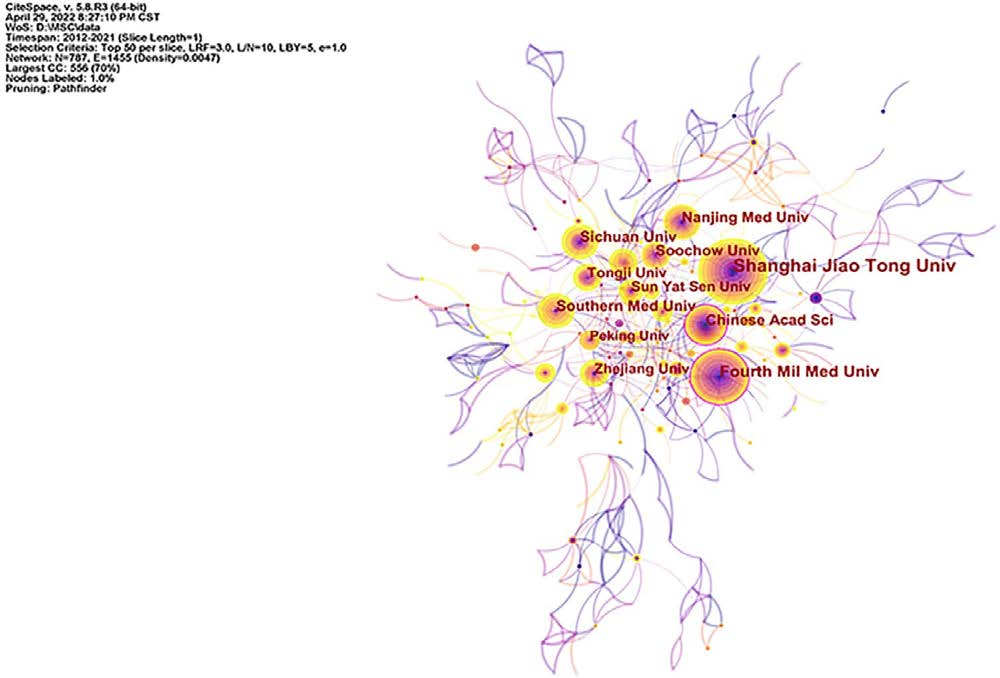
Knowledge map of institution collaboration.

By analyzing the cooperation network of institutions in this field, a knowledge graph consisting of 787 nodes and 1455 connections was obtained, as shown in Figure 4. The top institutions in terms of published papers were Shanghai Jiaotong University (109 papers), Fourth Military Medical University (65 papers), Sichuan University (50 papers), Chinese Academy of Sciences (48 papers) and Southern Medical University (45 papers), etc. The top institutions for centrality were Chinese Academy of Sciences (0.13), Fourth Military Medical University (0.1), Sun Yat-sen University (0.09), Shanghai Jiaotong University (0.08) and Southern Medical University (0.08), etc. It can be seen from the results that most of the institutions with the highest publication volume and centrality are Chinese institutions of higher learning, indicating that Chinese scholars have ranked first in the world in terms of publication volume in the past 10 years, and domestic institutions had been closely cooperating.

### 3.5. Countries

**Figure 5.**
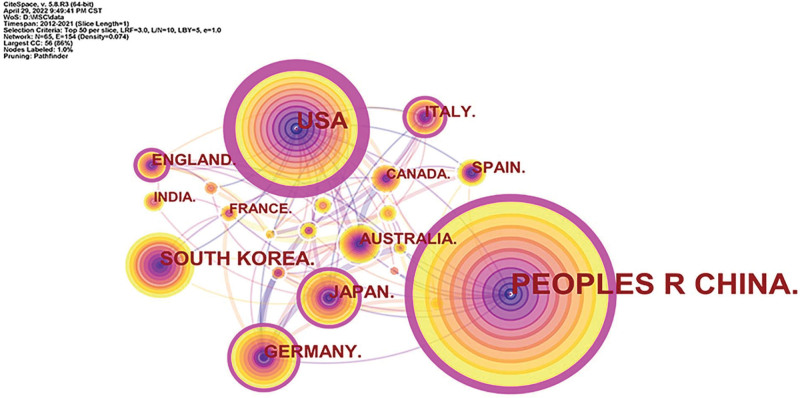
Knowledge map of country collaboration.

The national cooperation network in this field was analyzed, and a knowledge graph consisting of 65 nodes and 154 connections was obtained, as shown in Figure 5. The countries with the highest number of published articles were China (1247 articles), the United States (392 articles), South Korea (102 articles), Germany (80 articles) and Japan (68 articles), etc. The countries with the highest centrality ranking were United States (0.59), China (0.22), Italy (0.18), United Kingdom (0.16) and Germany (0.12). China led other countries in the number of published papers, but there was still a certain gap between the centrality and the United States.

### 3.6. Co-citations references

**Figure 6.**
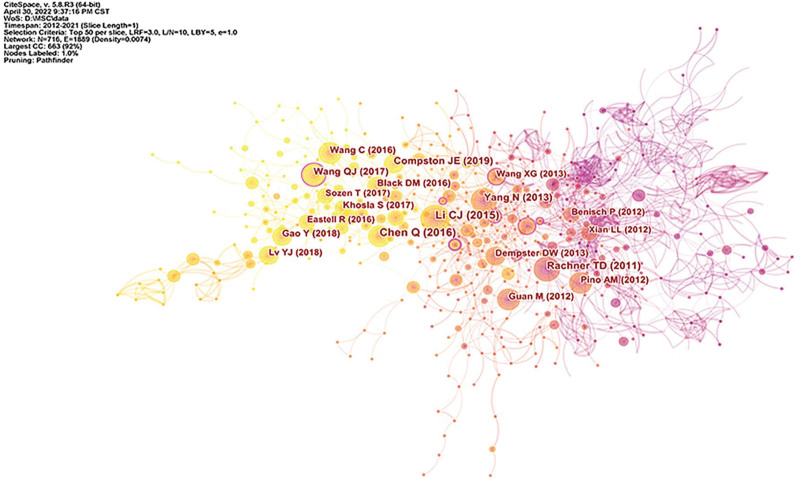
Knowledge map of co-citation references.

After analyzing the total citations of the literature in this field, a knowledge graph consisting of 719 nodes and 1889 connections was obtained, as shown in Figure 6. The specific information of the top 10 literatures ranked by citation frequency and centrality was shown in Table 1. Co-citation means that two articles are cited by the same citing document. The larger the node, the more citations. The thicker the line between the two nodes means the greater the co-citation strength. The higher the number of citations, the more common research topic.^[12]^ Through visual analysis, two articles with high citation frequency and high centrality were found. Li et al^[13]^ found that miR-188 was a key regulator of MSCs switching between age-related osteogenesis and adipogenesis, and may be a potential target for the treatment of age-related bone loss. Wang et al^[14]^ found through experiments that these data suggest that miR-214 has an important role in inhibiting osteogenesis, and inhibiting miR-214 in osteoblasts may be a potential anabolic strategy for improving OP.

**Table 1:** Top 10 co-cited references ranked by the number of citations and centrality.

Rank	First author	Year	Citations	Rank	First author	Year	Centrality
1	Li CJ	2015	58	1	Wang XG	2013	0.15
2	Chen Q	2016	55	2	Wang QJ	2017	0.14
3	Rachner TD	2011	49	3	Liao L	2013	0.13
4	Yang N	2013	41	4	Li CJ	2015	0.1
5	Compston JE	2019	40	5	Benisch P	2012	0.09
6	Wang QJ	2017	38	6	Chen Q	2016	0.08
7	Black DM	2016	33	7	Gao Y	2018	0.07
8	Wang C	2016	33	8	Qi M	2017	0.07
9	Guan M	2012	33	9	Rachner TD	2011	0.06
10	Khosla S	2017	32	10	Yang N	2013	0.04

### 3.7. Keywords

#### 3.7.1. Co-occurrence analysis.

**Figure 7.**
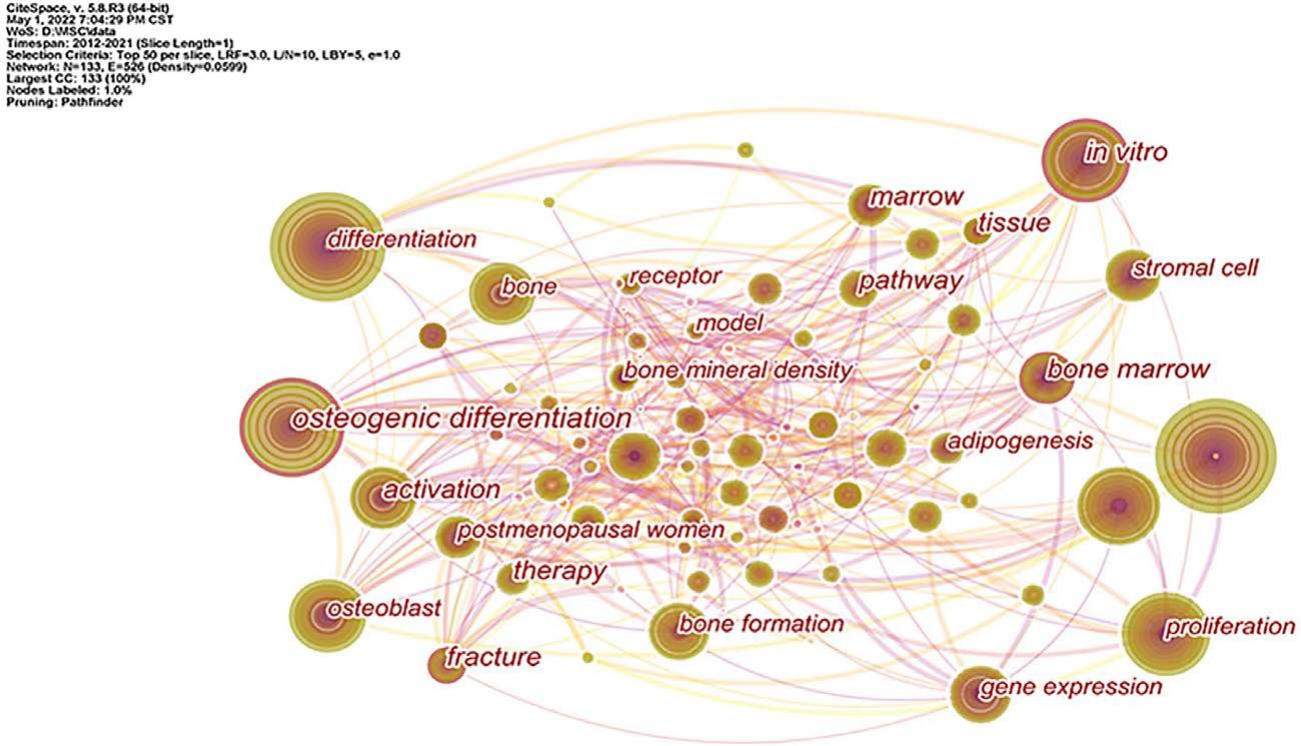
Knowledge map of keywords co-occurrence.

After analyzing the keywords in this field, a knowledge graph consisting of 133 nodes and 526 connections was obtained, as shown in Figure 7. Remove the subject words “mesenchymal stem cell,” “osteoporosis,” etc. related to the retrieval strategy, “differentiation” had the highest frequency with 413 times; “osteogenic differentiation” had the highest centrality with a value of 0.15. The specific information of the top 10 keywords with frequency and centrality was shown in Table 2. Keywords with high centrality and frequency represent research hotspots in a certain field in this time period.^[15]^ After analyzing the keywords, it is found that the research hotspots in this field mainly focus on the proliferation and osteogenic differentiation of MSCs,^[16–18]^ osteogenesis-related genes Expression and so on.^[19–21]^

**Table 2:** TOP 10 Keywords in terms of frequency and centrality.

Rank	Frequency	Keywords	Rank	Centrality	Keywords
1	413	differentiation	1	0.15	osteogenic differentiation
2	412	expression	2	0.12	bone marrow
3	394	osteogenic differentiation	3	0.11	fracture
4	250	in vitro	4	0.1	in vitro
5	239	proliferation	5	0.09	bone
6	232	osteoblast differentiation	6	0.09	bone formation
7	185	osteoblast	7	0.09	activation
8	165	bone	8	0.08	differentiation
9	158	bone formation	9	0.08	stromal cell
10	153	activation	10	0.08	tissue

#### 3.7.2. Cluster analysis.

**Figure 8.**
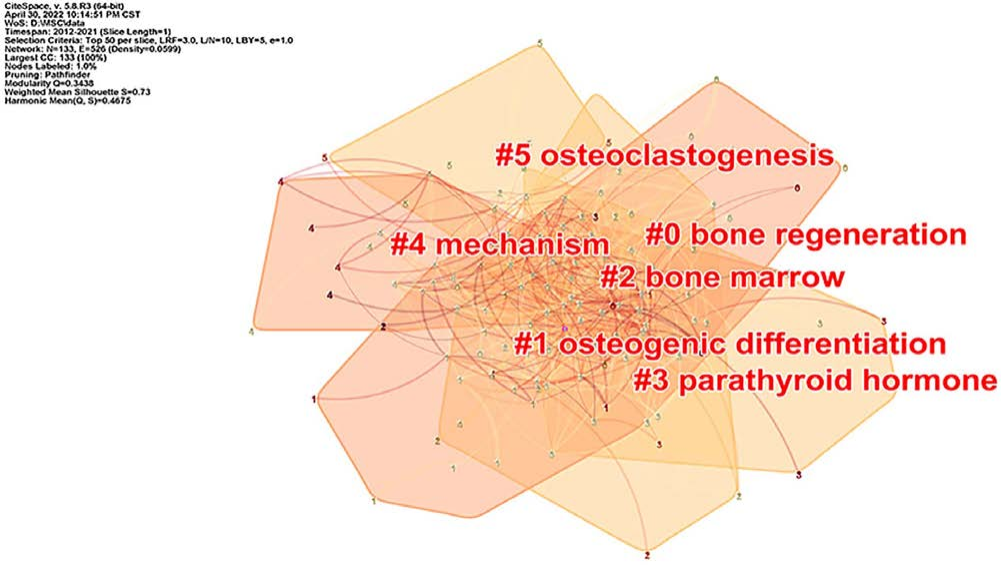
Knowledge map of keywords clusters.

**Figure 9.**
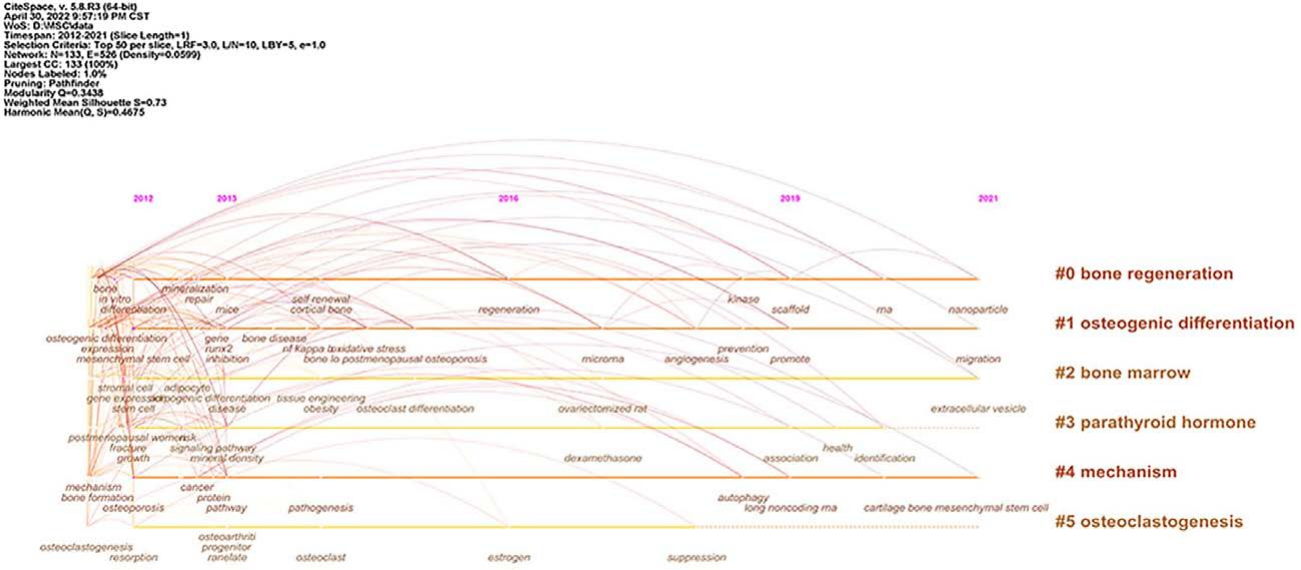
Timeline view of keywords.

The log-likelihood ratio (LLR) algorithm was applied to perform cluster analysis on the keywords, as shown in Figure 8. The silhouette value of cluster groups was 0.73, indicating that the clustering result was credible. A total of 6 clusters were obtained, which were #0 bone regeneration, #1 osteogenic differentiation, #2 bone marrow, #3 parathyroid hormone, # 4 mechanism, #5 osteoclastogenesis. The details of the clustering were shown in Table 3, and the time-line diagram of the clustering is shown in Figure 9.

**Table 3:** Keywords clustering information.

Cluster number	Size	Silhouette	Mean year	Cluster label
0	31	0.686	2013	bone regeneration
1	29	0.769	2013	osteogenic differentiation
2	25	0.713	2013	bone marrow
3	22	0.645	2013	parathyroid hormone
4	18	0.811	2014	mechanism
5	8	0.862	2013	osteoclastogenesis

#### 3.7.3. Burst analysis.

**Figure 10.**
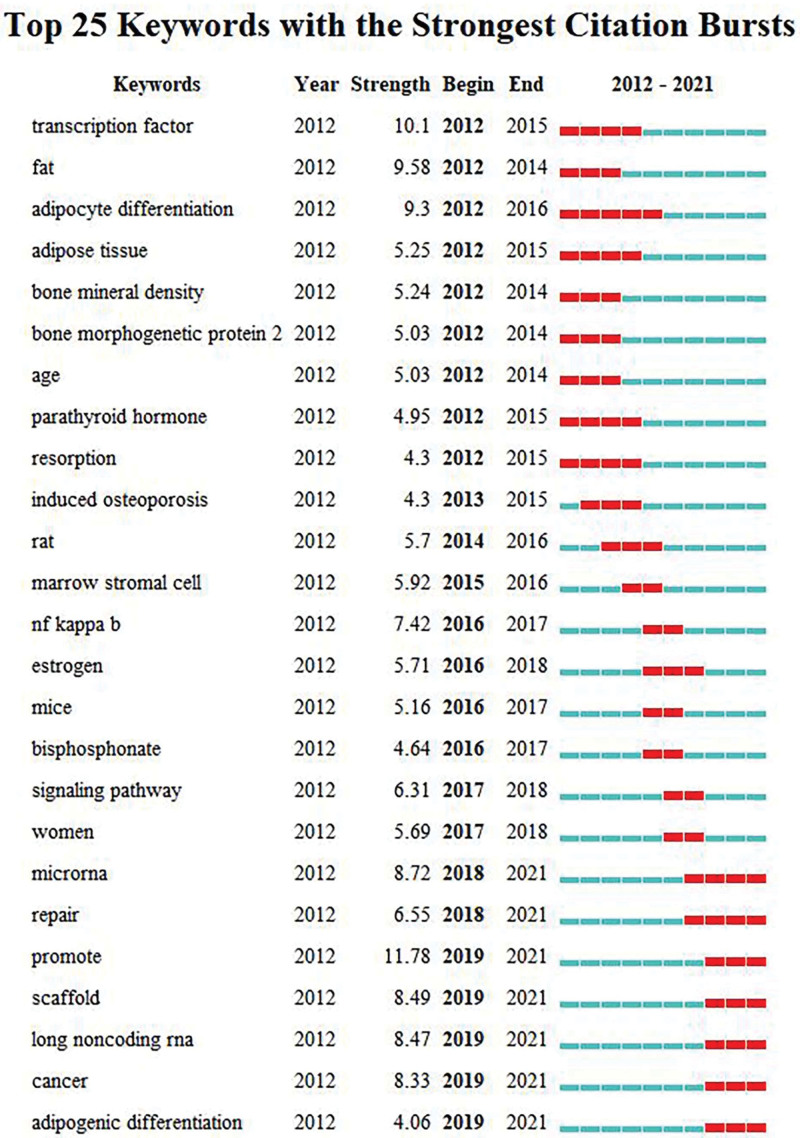
Top 25 keywords with the strongest citation bursts.

Burst words are keywords with high frequency change rate in a certain period of time, which can show the development trend of a certain research field to a certain extent.^[22]^ Burst analysis was performed on the keywords, and the top 25 burst word maps of the emergent intensity were obtained, as shown in Figure 10. According to the analysis results, the research in this field can be divided into three stages. The first stage was from 2012 to 2015, mainly focusing on transcription factors,^[23]^ adipocyte differentiation,^[24]^ bone morphogenetic proteins,^[25]^ parathyroid hormone^[26]^ and other mechanisms that lead to the occurrence of OP and the pathogenesis of senile OP.^[27]^ The second stage is from 2015 to 2018, mainly focusing on the role of NF-KB (nuclear factor kappa-B, NF-KB) and other signaling pathways in OP,^[28,29]^ and the therapeutic effect of bisphosphonates and estrogen on OP^[30,31]^ and related studies of postmenopausal OP in women.^[21]^ The third stage was from 2018 to 2021, focusing on the mechanism of action of microRNA and long non-coding RNA on OP,^[20,32,33]^ the regulatory mechanism of MSCs osteogenic and adipogenic differentiation on OP^[24,34,35]^ and The application of stents in the treatment of OP.^[36]^

## 4. Discussion

### 4.1. Research status and development trend

The research in this paper shows that from 2012 to 2021, the number of published papers in this field has grown steadily, which can be divided into three stages. From 2012 to 2014, the growth rate was small, and the upward trend was not obvious. From 2014 to 2016, the number of published articles increased sharply, showing a rapid development trend. From 2016 to 2021, the annual growth rate remained stable, showing a steady upward trend. The average annual publication volume was 210, which showed that the research prospect in this field is good and the development space is broad. From the analysis of the author’s cooperation network, the author with the most publications was YAN JIN, whose main research direction was the effects of tumor necrosis factor and autophagy on MSCs and OP.^[37,38]^ The author with the highest centrality was BO GAO, whose main research direction was the regulation of Wnt signaling pathway and melatonin on MSCs and the effect on OP.^[39–41]^ The analysis found that the author centrality in this field is generally low, and the authors with no centrality > 0.1, to a certain extent, indicated that the research in this field was still in the exploratory stage. From the analysis of the journal cooperation network, the publication volume and centrality of *J BONE MINER RES* ranked first, and the influence was greater. From the perspective of institutional cooperation network analysis, the institution with the largest number of publications was Shanghai Jiaotong University. The institution with the highest centrality was the Chinese Academy of Sciences. From the analysis of the national cooperation network, China and the United States ranked first and second respectively in the number of documents published, and the number of documents published by China far exceeded that of the United States and other countries. However, the centrality of the United States was the highest, indicating that the United States had more in-depth research in this field and had academic influence.

Through the keyword co-occurrence analysis, it could be seen that differentiation, expression, osteogenic differentiation, in vitro, proliferation, osteoblast differentiation, osteoblast bone cells, bone, bone formation, activation and other keywords were hot keywords in this field. Through keyword clustering analysis, a total of 6 clusters were obtained. The smaller the cluster number, the more keywords the cluster contains. #1 bone regeneration, #2 osteogenic differentiation, #3 bone marrow, #4parathyroid hormone, #5 mechanism, #6 osteoclastogenesis, a total of 6 clusters were the research hotspots in this field. Combined with the keyword clustering timeline diagram, it can be seen that bone regeneration, osteogenic differentiation, bone marrow and mechanism will remain the research hotspots in 2021. Therefore, the research hotspots of MSCs in the treatment of OP mainly focused on the mechanism of bone regeneration, proliferation and osteogenic differentiation of bone marrow MSCs, and the expression of osteogenic-related genes.

According to the burst analysis of keywords, the development trend of this field can be inferred. From 2018 to 2021, there are 7 burst words, namely: microRNA, repair, promote, scaffold, long noncoding RNA, cancer, adipogenic differentiation. Therefore, the future development trend in this field mainly focuses on the mechanism of microRNA and long non-coding RNA on MSCs and their relationship with OP, the mechanism of MSCs adipogenic and osteogenic differentiation, and the application of tissue engineering scaffolds.

### 4.2. Limitations

This paper analyzed the relevant content of this field through the application of CiteSpace software, and understood the overall research situation in this field. However, this paper only analyzed the literature from WOSCC database, and didn’t analyze other databases, and the analysis results had certain limitations.

### 4.3. The significance of the research

This article included the recent 2012 to 2021 literature on the treatment of OP with MSCs in the WOSCC database. The application of CiteSpace software for bibliometric and visual analysis could help scholars understand the research status, hotspots and development trends in this field, and provide some reference for their research on the application of MSCs in the treatment of OP.

## Author contributions

**Data curation:** Yueying Wang.

**Investigation:** Ning Xu.

**Methodology:** Runfang Wang, Yueying Wang, Weiyi Zai.

**Software:** Weiyi Zai.

**Supervision:** Ning Xu.

**Writing – original draft:** Runfang Wang.

**Writing – review & editing:** Runfang Wang.

## References

[1] Ibrahim NA Nabil N Ghaleb S. Pathophysiology of the risk factors associated with osteoporosis and their correlation to the T-score value in patients with osteopenia and osteoporosis in the United Arab Emirates. J Pharm Bioallied Sci. 2019;11:364–72. 31619919 10.4103/jpbs.JPBS_4_19 PMC6791084

[2] Srivastava RK Sapra L. The rising era of “Immunoporosis”: role of immune system in the pathophysiology of osteoporosis. J Inflamm Res. 2022;15:1667–98. 35282271 10.2147/JIR.S351918 PMC8906861

[3] Eb A Uhm A Vnv A. The IFN-γ-mini/TrpRS signaling axis: an insight into the pathophysiology of osteoporosis and therapeutic potential. Cytokine Growth Factor Rev. 2022;64:7–11. 35115234 10.1016/j.cytogfr.2022.01.005

[4] Wang Y Yao J Cai L. Bone-targeted extracellular vesicles from mesenchymal stem cells for osteoporosis therapy. Int J Nanomedicine. 2020;15:7967–77. 33116512 10.2147/IJN.S263756 PMC7573321

[5] He XY Yu HM Lin S Li Y-Z. Advances in the application of mesenchymal stem cells, exosomes, biomimetic materials, and 3D printing in osteoporosis treatment. Cell Mol Biol Lett. 2021;26:47. 34775969 10.1186/s11658-021-00291-8 PMC8591870

[6] Lu CH Chen YA Ke CC Liu R-S. Mesenchymal stem cell-derived extracellular vesicle: a promising alternative therapy for osteoporosis. Int J Mol Sci . 2021;22:12750. 34884554 10.3390/ijms222312750 PMC8657894

[7] Shou X Wang Y Jia Q. Knowledge domain and emerging trends in Takotsubo cardiomyopathy: a scientometric review based on CiteSpace analysis. Ann Palliat Med. 2022;11:1505–17. 35400161 10.21037/apm-21-2645

[8] Luo H Cai Z Huang Y. Study on pain catastrophizing from 2010 to 2020: a bibliometric analysis via CiteSpace. Front Psychol. 2021;12:759347. 34975649 10.3389/fpsyg.2021.759347 PMC8718514

[9] Dang Q Luo Z Ouyang C. First systematic review on health communication using the CiteSpace Software in China: exploring its research hotspots and frontiers. Int J Environ Res Public Health. 2021;18:1300813008. 10.3390/ijerph182413008 PMC8702194 34948617

[10] Zhu K Lin R Li H. Study of virtual reality for mild cognitive impairment: a bibliometric analysis using CiteSpace. Int J Nurs Sci. 2021;9:129–36. 35079614 10.1016/j.ijnss.2021.12.007 PMC8766785

[11] Pei W Peng R Gu Y Zhou X Ruan J. Research trends of acupuncture therapy on insomnia in two decades (from 1999 to 2018): a bibliometric analysis. BMC Complement Altern Med. 2019;19:225. 31438914 10.1186/s12906-019-2606-5 PMC6704508

[12] Fu L Sun Z He L. Global long-term care research: a scientometric review. Int J Environ Res Public Health. 2019;16:2077-+. 10.3390/ijerph16122077 PMC6616636 31212782

[13] Li CJ Peng C Liang MK. MicroRNA-188 regulates age-related switch between osteoblast and adipocyte differentiation. J Clin Invest. 2015;125:1509–22. 25751060 10.1172/JCI77716 PMC4396470

[14] Wang X Guo B Li Q. miR-214 targets ATF4 to inhibit bone formation. Nat Med. 2013;19:93–100. 23223004 10.1038/nm.3026

[15] Guo Y Xu ZY Cai MT. Epilepsy with suicide: a bibliometrics study and visualization analysis via CiteSpace. Front Neurol. 2022;12:823474. 35111131 10.3389/fneur.2021.823474 PMC8802777

[16] Huang Y Zheng Y Jin C Li X Jia L Li W. Long non-coding RNA H19 inhibits adipocyte differentiation of bone marrow mesenchymal stem cells through epigenetic modulation of histone deacetylases. Sci Rep. 2016;6:28897. 27349231 10.1038/srep28897 PMC4924093

[17] Liu Y Berendsen AD Jia S. Intracellular VEGF regulates the balance between osteoblast and adipocyte differentiation. J Clin Investig. 2012;122:3101–13. 22886301 10.1172/JCI61209 PMC3428080

[18] Gao Y Xiao F Wang C. Long noncoding RNA MALAT1 promotes osterix expression to regulate osteogenic differentiation by targeting miRNA-143 in human bone marrow-derived mesenchymal stem cells. J Cell Biochem. 2018;12:823474. 10.1002/jcb.26907 29741283

[19] Zhu Y Tchkonia T Pirtskhalava T. The Achilles’ heel of senescent cells: from transcriptome to senolytic drugs. Aging Cell. 2015;14:644–58. 25754370 10.1111/acel.12344 PMC4531078

[20] Kelch S Balmayor ER Seeliger C Vester H Kirschke JS van Griensven M. MiRNAs in bone tissue correlate to bone mineral density and circulating miRNAs are gender independent in osteoporotic patients. Sci Rep. 2017;7:15861. 29158518 10.1038/s41598-017-16113-x PMC5696459

[21] Mandourah AY Ranganath L Barraclough R. Circulating microRNAs as potential diagnostic biomarkers for osteoporosis. Sci Rep. 2018;8:8421. 29849050 10.1038/s41598-018-26525-y PMC5976644

[22] Zhou Q Kong HB He BM Zhou S-Y. Bibliometric analysis of bronchopulmonary dysplasia in extremely premature infants in the web of science database using CiteSpace Software. Front Pediatr. 2021;9:705033. 34490163 10.3389/fped.2021.705033 PMC8417835

[23] Wijnen AJ Peppel J Leeuwen JP. MicroRNA functions in osteogenesis and dysfunctions in osteoporosis. Curr Osteoporos Rep. 2013;11:72–82. 23605904 10.1007/s11914-013-0143-6 PMC3678273

[24] Yang K Cao W Hao X. Metallofullerene nanoparticles promote osteogenic differentiation of bone marrow stromal cells through BMP signaling pathway. Nanoscale. 2013;5:1205–12. 23299786 10.1039/c2nr33575a

[25] Zhang Y Cheng N Miron R Shi B Cheng X. Delivery of PDGF-B and BMP-7 by mesoporous bioglass/silk fibrin scaffolds for the repair of osteoporotic defects. Biomaterials. 2012;33:6698–708. 22763224 10.1016/j.biomaterials.2012.06.021 PMC5995476

[26] Mei B Wang Y Ye W. LncRNA ZBTB40-IT1 modulated by osteoporosis GWAS risk SNPs suppresses osteogenesis. Hum Genet. 2019;138:151–66. 30661131 10.1007/s00439-019-01969-y

[27] Wang H Chen Q Lee SH Choi Y Johnson FB Pignolo RJ. Impairment of osteoblast differentiation due to proliferation-independent telomere dysfunction in mouse models of accelerated aging. Aging Cell. 2012;11:704–13. 22621437 10.1111/j.1474-9726.2012.00838.x PMC3400025

[28] Xia L Yin Z Mao L. Akermanite bioceramics promote osteogenesis, angiogenesis and suppress osteoclastogenesis for osteoporotic bone regeneration. Sci Rep. 2016;6:22005. 26911441 10.1038/srep22005 PMC4766478

[29] Chen X Zhi X Cao L. Matrine derivate MASM uncovers a novel function for ribosomal protein S5 in osteoclastogenesis and postmenopausal osteoporosis. Cell Death Dis. 2017;8:e3037. 28880271 10.1038/cddis.2017.394 PMC5636967

[30] Saito A Nagaishi K Iba K. Umbilical cord extracts improve osteoporotic abnormalities of bone marrow-derived mesenchymal stem cells and promote their therapeutic effects on ovariectomised rats. Sci Rep. 2018;8:1161. 29348535 10.1038/s41598-018-19516-6 PMC5773568

[31] Misra J Mohanty ST Madan S. Zoledronate attenuates accumulation of DNA damage in mesenchymal stem cells and protects their function. Stem Cells. 2015;34:756–67. 26679354 10.1002/stem.2255 PMC4832316

[32] Yang M Li CJ Sun X. MiR-497195 cluster regulates angiogenesis during coupling with osteogenesis by maintaining endothelial Notch and HIF-1α activity. Nat Commun. 2017;8:16003. 28685750 10.1038/ncomms16003 PMC5504303

[33] Gao Y Xiao F Wang C. Long noncoding RNA MALAT1 promotes osterix expression to regulate osteogenic differentiation by targeting miRNA-143 in human bone marrow-derived mesenchymal stem cells. J Cell Biochem. 2018;119:6986–96. 29741283 10.1002/jcb.26907

[34] Wang Q Li Y Zhang Y. LncRNA MEG3 inhibited osteogenic differentiation of bone marrow mesenchymal stem cells from postmenopausal osteoporosis by targeting miR-133a-3p. Biomed Pharmacother. 2017;89:1178–86. 28320084 10.1016/j.biopha.2017.02.090

[35] Huang JM Yuan B Wei X. Icariin regulates the bidirectional differentiation of bone marrow mesenchymal stem cells through canonical wnt signaling pathway. Evid Based Compl Altern Med. 2017;2017:8085325. 10.1155/2017/8085325 PMC5763109 29445413

[36] Zhang X Cui J Cheng L Lin K. Enhancement of osteoporotic bone regeneration by strontium-substituted 45S5 bioglass via time-dependent modulation of autophagy and the Akt/mTOR signaling pathway. J Mater Chem B. 2021;9:3489–501. 33690737 10.1039/d0tb02991b

[37] Yang N Wang G Hu C. Tumor necrosis factor α suppresses the mesenchymal stem cell osteogenesis promoter miR-21 in estrogen deficiency-induced osteoporosis. J Bone Miner Res. 2013;28:559–73. 23074166 10.1002/jbmr.1798

[38] Yang M Meng Q Ying A. Autophagy controls mesenchymal stem cell properties and senescence during bone aging. Aging Cell. 2018;17. 10.1111/acel.12709 PMC5770781 29210174

[39] Jing H Su X Gao B. Epigenetic inhibition of Wnt pathway suppresses osteogenic differentiation of BMSCs during osteoporosis. Cell Death Dis. 2018;9:176. 29416009 10.1038/s41419-017-0231-0 PMC5833865

[40] Lian C Wu Z Gao B. Melatonin reversed tumor necrosis factor-alpha-inhibited osteogenesis of human mesenchymal stem cells by stabilizing SMAD1 protein. J Pineal Res. 2016;61:317–27. 27265199 10.1111/jpi.12349

[41] Qiu X Wang X Qiu J. Melatonin rescued reactive oxygen species-impaired osteogenesis of human bone marrow mesenchymal stem cells in the presence of tumor necrosis Factor-Alpha. Stem Cells Int. 2019;2019:6403967. 31582985 10.1155/2019/6403967 PMC6754961

